# Reliability test and revision of stress coping scale for early childhood teachers

**DOI:** 10.3389/fpsyg.2024.1501478

**Published:** 2025-01-06

**Authors:** Jie Wang

**Affiliations:** School of Education, Zhanjiang University of Science and Technology, Zhanjiang, Guangdong Province, China

**Keywords:** early childhood teachers, stress coping, scales, reliability, validity

## Abstract

The purpose of this research was to determine the reliability and validity of the Early Childhood Teachers’ Stress Coping Strategies Scale measured among early childhood teachers in Guangdong Province, China. The researcher used the Early Childhood Teachers’ Stress Coping Strategies Scale and a simplified version of the COPE questionnaire (Coping Inventory). The Early Childhood Teachers’ Stress Coping Strategies Scale consists of three dimensions: “Rational Thinking,” “Emotional Regulation,” and “Support Seeking,” and the simplified COPE questionnaire was consists of 2 dimensions: “Help Seeking” and “Cognitive Coping.” The scale was administered to 653 in-service ECE teachers and retested with a sample of 320 ECE teachers 1 month later. The internal consistency coefficient (ICC) of the questionnaire as a whole was 0.91, the internal consistency reliabilities of the 3 subscales ranged from 0.86–0.91, and the ICC values of the intragroup correlation coefficients of the latent variables ranged from 0.864–0.881, with a *p* < 0.05, which showed good reliabilities. Validity: exploratory factor analysis extracted a total of three factors with characteristic roots greater than 1. The three factors were denominated as, Rational Thinking, Emotion Regulation, and Support Seeking, and the three factors explained a total of 53.82% of the variance. The validation factor analysis showed that the model fit indices were GFI > 0.85, CFI > 0.9, NFI > 0.85, TLI > 0.9, IFI > 0.9, RMSEA<0.10, and RMR = <0.05, which indicated that the coping strategy scale with a good structural stability. Among the three-factor model, the discriminant and convergent validity of the three factors were reasonable. The correlation coefficients of rational thinking, emotion regulation, and support seeking with the two dimensions of help-seeking and cognitive coping in the COPE questionnaire were significantly positive and greater than 0.4, indicated that the correlation between this scale and the standardized scales was high. Overall, the Early Childhood Teachers’ Stress Coping Strategies Scale could be used for the measurement of early childhood teachers’ stress coping strategies.

## 1 Introduction

Since young children entered their first early childhood education institution, the first adult they came into with and directly interacted continuously was the early childhood teachers (Liu, 2022). The preschool period of 3–6 years old is a vital period for young children to build up trust with others and develop social skills, therefore, the career stability of early childhood teachers is closely related to the development of young children (Song, 2019), as frequent change of teachers can lead to increased separation anxiety in young children, as evidenced by crying, violence, or indifference, and indirectly impair their personality development and social behavior (Friedman-Krauss et al., 2014). Numerous studies have shown that the career stability of early childhood teachers was associated with their perceived occupational stress in different degrees (Wang, 2023; Cao and Yang, 2022; Wang et al., 2022), and that as the level of occupational stress increased, the career stability of early childhood teachers decreased, and their resignation intention increased.

Occupational stress had become a common occurrence in the field of pre-school education as the social requirements increased and the stress level was on the rise, however, not all early childhood teachers in similar occupational environments felt great stress and had a desire to leave their jobs as a result. The majority of teachers remain with a normal mental health status and continue to work.

In earlier studies, factors affecting the career stability of early childhood teachers focused on demographic variables such as teachers’ age, income, and marital status (Zhang, 2024; Sheng, 2020). With the expansion of research, the factors affecting the occupational stability of early childhood teachers have increased, and researchers have focused on external factors such as social support (Wang, 2022), social expectations (Wu, 2023), and interpersonal relationships (Li, 2022) and internal factors such as teachers’ psychological resilience (Zeng, 2022), learning ability, and teachers’ coping strategies for stress, which all affect the occupational stability of teachers.

After controlling for variables such as income, age, and gender, there were differences in coping strategies and the ability to use coping strategies for occupational stress among teachers in similar occupational settings (Tseng, 2018); teachers who successfully coped with stress performed well on the job, however, those with a lack of coping strategies usually worked poorly and eventually left their jobs due to overwhelm. Researchers came to realize that coping strategies may be an important variable that affects occupational stability, thus exploring coping strategies for occupational stress among early childhood teachers is a new direction for the future of the preschool field.

The current research literature on the measurements of stress coping strategies of early childhood teachers is not rich enough, and one of the possible reasons for this may be due to the lack of appropriate measurement tools (Lee, 2018). Currently, the dominant way of collecting data in the field of educational psychology is to invite subjects to fill in scales and questionnaires to obtain relevant data. In the current literature, researchers have used different instruments to measure stress coping strategies, such as the 66-item WCQ (Ways of Coping Questionnaire) developed by Lazarus and Folkman (1984), Carver and Darling’s COPE questionnaire (Coping Inventory) based on “problem-oriented” and “emotion-oriented” theories (Carver-Thomas and Darling-Hammond, 2017), Xiao and Xu’s coping questionnaire (Xiao and Xu, 1996), and Huang’s Coping Strategies Scale (Wong, 2004).

The COPE questionnaire contains 28 items, which are divided into two dimensions of “help-seeking” and “cognitive coping,” and each subscale consists of 12 items. The translation and revision of the simplified version of the COPE questionnaire by Xiong showed that the internal consistency *α* of the scale was 0.78 (Xiong, 2008), but it did not show the reliability of each scale, while the questionnaire developed by Xiao and Xu covers 6 factors with 62 items. In the sample of college students, the re-test reliability questionnaire developed by Xiao and Xu was 0.72, 0.62, 0.69, 0.72, 0.67, and 0.72, and the Coping Strategies Scale (CSS) developed by Huang with reference to the instruments developed by the scholars in China. It was designed to understand the coping strategies of kindergarten teachers in the face of occupational stress, and the Cronbach’s *α* coefficients of the three dimensions of coping strategies, namely rational thinking, emotion regulation and support seeking were 0.95, 0.82, 0.88 in order of magnitude, and 0.94 for the whole scale.

After comparison, it was found that some of the measurement tools had a large number of questions and it was not clear what kind of target subjects the scales were developed for. Scales with too many questions could easily lead to fatigue of the respondents, which would affect the accuracy and validity of the data, and scales that were not clear what kind of target subjects the scales were developed for might not have a good reliability and validity when they were applied to different groups of people, and some of the reliability and validity of the measurement tools need to be improved. Among the existing research tools, Huang’s scale has a Cronbach’s *α* value greater than 0.8 and a smaller number of questions, which makes it more convenient to use.

Huang’s scale is based on the Cognitive Interaction Theory presented by Lazarus and Folkman in 1984, which suggests that stress and coping are dynamic processes of cognitive appraisal that can be divided into five steps: ① judgment of whether an event is stressful; ② primary cognitive appraisal of the event; ③ secondary appraisal; ④ choice of coping style; and ⑤ adjustment of coping styles until adapting to the outcome. Cognitive interaction theory emphasizes that cognitive assessment is a constantly repeating process, and individuals will adopt coping styles according to the assessment results. Therefore, cognitive assessment is the basis for determining whether an individual is under stress and how to cope with it. Huang’s scale categorizes coping strategies into three dimensions 1. Rational Thinking: Understanding the source of stress and evaluating the possible consequences, rationally analyzing coping methods to make the situation favorable. 2. Support Seeking: Talking to experienced people or friends and relatives, seeking solutions to problems or moral support to relieve stress. 3. Emotional Adjustment: Adjust emotions and actively participate in leisure activities to relieve emotions.

Huang’s scale was widely used in the literature of preschool or primary education in mainland China and Taiwan, and it was also used by researchers as a reference for the development of other coping strategy scales (Darius et al., 2021; Yang, 2021; Bian, 2018; Cao et al., 2021; Cai, 2021), but subsequent users did not re-test its reliability and validity in their respective dissertations. Therefore, the validity of Wong (2004) scale can assist in proving the validity and reliability of the results of the study. Accordingly, the researcher selected kindergarten teachers in Guangzhou City as the study population and verified the validity and reliability of the Early Childhood Teachers’ Stress Coping Strategies Scale by observing the Cronbach’s *α* coefficient, convergent validity, convergent validity, and model fit index.

## 2 Research methodology

### 2.1 Research instruments

This study is a non-experimental study, the subjects of the study are in-service kindergarten teachers in Guangzhou City, and the researcher obtained the data by using a survey questionnaire. For this reason, the scales used in this research were Early Childhood Teachers’ Stress Coping Strategies Scale developed by Huang and the COPE Simple Version translated by Xiong. The Early Childhood Teachers’ Stress Coping Strategies Scale developed by Huang was applied to in-service early childhood teachers and was mainly used to measure the level of stress coping strategies of early childhood teachers. The questionnaire consists of 24 questions divided into 3 dimensions. The whole scale was scored on a 5-point Likert-type scale, in which respondents selected their answers according to their personal situation, ranging from “very much in line with” (5 points) to “very much not in line with” (1 point), and the higher the score, the more often the strategy was used. Higher scores indicated more frequent use of the strategy; lower scores indicated less frequent use of the strategy.

The simplified version of the COPE questionnaire translated by Xiong was used as a standardized scale to test the validity of the target scale, which has been widely used in the literature on stress coping strategies. The Chinese version of the COPE questionnaire consists of 13 items, and the whole scale is scored on a Likert-type 4-point scale, which consists of instrumental coping, help-seeking, cognitive coping, emotional catharsis, and drug utilization, in which the dimensions of emotional catharsis and drug utilization have only one item, which is less than the requirement of the measurement standard of 3 items, and some of the questions are not suitable for early childhood teachers due to the difference in cultural backgrounds, therefore, the dimensions of seeking help, cognitive coping were selected to be measured. so the two dimensions of help-seeking and cognitive coping were selected for measurement.

### 2.2 Population

The target population of this research is all in-service kindergarten teachers in Guangzhou and Zhanjiang City, China. This study used stratified sampling method to select one public and one private kindergarten from 11 districts in Guangzhou and 4 districts in Zhanjiang City according to the actual situation, and then invited 20% of kindergarten teachers from each kindergarten to fill in the questionnaires as samples, and a total of 700 questionnaires were distributed, of which 653 completed the questionnaire survey. A total of 653 teachers completed the questionnaire. Eighty six male teachers and 567 female teachers participated in the survey. The mean age of the teachers tested was 24.13 years (M = 24.13, SD = 3.85).

### 2.3 Data collection

The researcher invited teachers to fill in the questionnaire by scanning the QR code with the consent and support of each director. Before filling out the questionnaire, teachers read the purpose, principles and sign informed consent, and instructions on the first page, and then answered the questionnaire after agreeing to do so. Each teacher completed the questionnaire independently according to the introduction on the invitation letter. The researcher compiled statistics 1 week after the questionnaire was distributed. After excluded unfinished questionnaires and abnormal questionnaires with identical options, 653 valid questionnaires were obtained, and the recovery rate of valid questionnaires was 93.3%. One month later, teachers with dual-numbered questionnaires were invited to complete the questionnaires again for retesting, and 320 valid questionnaires were obtained after excluded the abnormal questionnaires that were not completed and had exactly the same choices, with a valid recovery rate of 98%.

### 2.4 Data analysis

After obtained the data through the questionnaire, the researcher conducted a measurement study on the Early Childhood Teachers’ Stress Coping Strategies Scale, include Cronbach’s *α* coefficient and retest reliability, model fit index, convergent validity and discriminant validity of the scale. Cronbach’s α coefficient greater than 0.8 was considered as a good fit. Model fit indices: GFI > 0.9, CFI > 0.9, NFI > 0.9, TLI > 0.9, IFI > 0.9 was considered as good model fit, AVE > 0.5 and CR > 0.7, indicated high convergent validity, AVE square root value was greater than the maximum of the absolute value of the correlation coefficient between the factors was considered as good discriminant validity, and the Early Childhood Teachers’ Stress Coping Strategies Scale was correlated to the standardized questionnaire to observe the correlation, and the correlation coefficient was significant (*p* < 0.05), then the correlation of the school standard was good.

## 3 Results

### 3.1 Descriptive statistics and reliability analysis

The descriptive statistics of the Early Childhood Teachers’ Stress Coping Strategies Scale was conducted, and the mean value and SD for the 24 items were presented in Table 1.

**Table 1:** Descriptive statistics of Early Childhood Teachers’ Stress Coping Strategies Scale.

Items	Mean	SD
1 Thinking about things from different perspectives	3.018	0.376
2 Negotiate to make the situation favorable	3.061	0.634
3 Refer to previous experience	2.975	0.646
4 Respecting different opinions	2.942	0.587
5 Reflect on teaching	2.908	0.611
6 Reviewing yourself	2.880	0.593
7 Think about the causes of problems	2.798	0.653
8 Reflect on yourself and analyze others	2.776	0.648
9 Consider consequences before communicating with parents	2.647	0.707
10 Prepare for the worst	2.791	0.571
11 Participate in activities to increase knowledge	3.294	0.520
12 Engage in artistic activities	3.132	0.696
13 Manage emotions well	3.141	0.837
14 Engage in sports	3.224	0.797
15 Engage in recreational activities	3.147	0.832
16 Build up from frustration	3.006	0.773
17 Keep myself busy	3.061	0.733
18 I will seek the cooperation of parents	2.724	0.783
19 Seek help from friends	2.926	0.777
20 Seek help from experienced people or groups	2.748	0.783
21 Seek help from superiors	2.626	0.797
22 Seek advice from education experts and scholars	2.304	1.364
23 Seek assistance and advice from other teachers	2.730	0.765
24 Seek assistance from professionals outside the school	2.414	0.889

The results of the descriptive statistics showed that the mean values of the coping strategies of the subjects ranged between 2.414 and 3.294, which was a moderate level.

The reliability analysis of the Early Childhood Teachers’ Stress Coping Strategies Scale was conducted, and the Cronbach’s *α* coefficients for the 24 items were presented in Table 2.

**Table 2:** Reliability analysis of Early Childhood Teachers’ Stress Coping Strategies Scale.

Factors	Cronbach’s α	Items
Rational thinking	0.884	11
Emotional regulation	0.862	6
Support seeking	0.871	7
Over all	0.912	24

Table 2 showed that the Early Childhood Teachers’ Stress Coping Strategies Scale and its dimensions Cronbach’s α coefficients ranged from 0.862–0.912, which were all greater than 0.8, with good reliability.

### 3.2 Validity analysis

Exploratory factor analysis was conducted on the Early Childhood Teachers’ Stress Coping Strategies Scale, analyzed by principal axis factor analysis and rotated with the use of the maximum variance method.

The exploratory factor analysis extracted three factors with characteristic root greater than 1. The three factors were named as rational thinking, emotional regulation, and support seeking, and the three factors explained 53.82% of the variance.

The data were further analyzed for validity and the indicators were observed to determine their validity. A total of 653 valid questionnaires were recovered in this study, the factors and the number of questions are shown in Table 3.

**Table 3:** Summary of factors and number of items.

Factors	Number of items
Rational thinking	11
Emotional regulation	6
Support seeking	7
Over all	24

As shown in Table 3, the Early Childhood Teachers’ Occupational Stress Coping Strategies Scale had 3 factors with 24 items. The valid sample size of this validated factor analysis was 653, which exceeded the number of analyzed items by 20 times, and the sample size was reasonable. The factor loading coefficients of the 24 question items are shown in Table 4.

**Table 4:** Factor loading coefficients.

Factors	Measured items (variable)	*p*	Std. Estimate
Rational thinking	1 Thinking about things from different perspectives	–	0.488
Rational thinking	2 Negotiate to make the situation favorable	0.000	0.764
Rational thinking	3 Refer to previous experience	0.000	0.491
Rational thinking	4 Respecting different opinions	0.000	0.638
Rational thinking	5 Reflect on teaching	0.000	0.702
Rational thinking	6 Reviewing yourself	0.000	0.694
Rational thinking	7 Think about the causes of problems	0.000	0.657
Rational thinking	8 Reflect on yourself and analyze others	0.000	0.711
Rational thinking	9 Consider consequences before communicating with parents	0.000	0.728
Rational thinking	10 Prepare for the worst	0.000	0.785
Rational thinking	11 Participate in activities to increase knowledge	0.000	0.742
Emotional regulation	12 Engage in artistic activities	–	0.625
Emotional regulation	13 Manage emotions well	0.000	0.767
Emotional regulation	14 Engage in sports	0.000	0.779
Emotional regulation	15 Engage in recreational activities	0.000	0.838
Emotional regulation	16 Build up from frustration	0.000	0.752
Emotional regulation	17 Keep myself busy	0.000	0.464
Support seeking	18 I will seek the cooperation of parents	–	0.761
Support seeking	19 Seek help from friends	0.000	0.757
Support seeking	20 Seek help from experienced people or groups	0.000	0.811
Support seeking	21 Seek help from superiors	0.000	0.792
Support seeking	22 Seek advice from education experts and scholars	0.000	0.453
Support seeking	23 Seek assistance and advice from other teachers	0.000	0.819
Support seeking	24 Seek assistance from professionals outside the school	0.000	0.681

The data in Table 4 show that the significance of all items is less than 0.01, and the absolute values of the standardized loading coefficients of the 24 items were between 0.453–0.838, which were all greater than 0.4 indicated that the correlation between the 24 items and the factors was high, and that all the items could be retained. The AVE (Average Variance Extraction) and CR (Combined Reliability) were used in the analysis of the Aggregate Validity (Convergent Validity), the results of which were presented in the Table 5.

**Table 5:** Aggregation validity: model AVE and CR metrics results.

Factors	AVE	CR
Rational thinking	0.503	0.902
Emotional regulation	0.511	0.861
Support seeking	0.543	0.890

Table 5 shows that the CR values corresponding to the three factors were greater than 0.7, and the average variance AVE values of the three factors were slightly higher than 0.5, implied that their convergent validity was still acceptable, but could still be optimized.

For the analysis of discriminant validity, for rational thinking, its AVE square root value was 0.697, which was slightly higher than the maximum value of the absolute value of the inter-factor correlation coefficient of 0.695, which means that its discriminant validity was still good but there was room for optimization. For Emotion Regulation and Support Seeking, the AVE square root values were 0.725 and 0.737, which were greater than the maximum absolute values of the correlation coefficients between the factors of 0.425 and 0.695, indicated that the two factors had good discriminant validity (Table 6).

**Table 6:** Potential variable correlations and AVE square root values.

	Rational thinking	Emotional regulation	Support seeking
Rational thinking	0.697		
Emotional regulation	0.532	0.725	
Support seeking	0.695	0.425	0.737

With reference to Lu′s study on the application of validated factor analysis in questionnaire development, Amos 21.0 was used to conduct validated factor analysis to explore the structural stability of the Early Childhood Teachers’ Occupational Stress Scale. Structural equation modeling analysis showed that the fit indices of the scale were shown in Table 7.

**Table 7:** Model fit indicators for the early childhood teachers’ occupational stress coping strategies scale.

χ^2^/df	RMSEA	RMR	CFI	NNFI	TLI	IFI	SRMR
2.400	0.066	0.034	0.914	0.905	0.905	0.915	0.063

The fit indices of the model showed fair structural stability of the coping strategy scale.

According to Table 8, it can be learned that the MI index of the item “Keeping yourself busy” was greater than 20 in both rational thinking and support seeking, and the item can be considered to be deleted because of possible cross-loading.

**Table 8:** Factors and measurement items - mi indicators.

Measurement items	Factors	MI value
Engaging in artistic activities	Rational thinking	15.124
Engage in sports	Rational thinking	17.820
Keep yourself busy	Rational thinking	23.581
Engage in artistic activities	Support seeking	11.271
Engage in recreational activities	Support seeking	10.961
Keep yourself busy	Support seeking	31.321

One month after the data collection, the researcher invited 320 teachers to retest, and correlated the retest data with the original scale data, and the results were shown in Table 8.

The 320 teachers were retested on the Stress Coping Strategies Scale after 1 month, and the results showed that the intragroup correlation coefficient ICC values for each latent variable ranged from 0.86–0.88, with a significance *p*-value of 0.000 < 0.01, which showed that the results of the test were highly reproducible, indicated that the scale had good retest reliability.

### 3.3 Correlation of the standardized scales

The three dimensions of the coping strategy scale Rational Thinking, Emotion Regulation, and Support Seeking were correlated with the COPE questionnaire, and the results were shown in Table 9.

**Table 9:** Correlations between the Early Childhood Teachers’ Stress Coping Strategies Scale and the standardized scale.

	Rational thinking	Emotional regulation	Support seeking	Help seeking	Cognitive coping
Rational thinking	–				
Emotional regulation	0.546**	–			
Support seeking	0.704**	0.433**	–		
Help seeking	0.725**	0.464*	0.473**	–	
Cognitive coping	0.617**	0.491**	0.598*	0.466**	–

**Means the significant correlation at the 0.01 level (two-tailed).

The results in Table 10 showed that Rational Thinking, Emotion Regulation, and Support Seeking were all significantly and positively correlated with the Help Seeking and Cognitive Coping dimensions of the COPE questionnaire, and the correlation coefficients were all greater than 0.4, which indicates that the scale is highly correlated with the standardized scales.

**Table 10:** Retest reliability ICC.

Variables	ICC	*P*	95% CI
Rational thinking	0.881	0.000	0.837–0.925
Emotional regulation	0.855	0.000	0.812–0.898
Support seeking	0.864	0.000	0.821–0.907

** Significant correlation at the 0.01 level (two-tailed).

The researcher used the revised scale to measure the level of coping strategies of the teachers and the results are presented in Table 11.

**Table 11:** Revised Early Childhood Teachers’ Stress Coping Strategies Scale descriptive statistics.

Items	Mean	SD
2 Negotiate to make the situation favorable	3.115	0.622
4 Respecting different opinions	2.913	0.529
5 Reflect on teaching	2.944	0.625
6 Reviewing yourself	3.016	0.422
7 Think about the causes of problems	2.887	0.671
8 Reflect on yourself and analyze others	2.725	0.609
9 Consider consequences before communicating with parents	2.718	0.724
10 Prepare for the worst	2.644	0.553
11 Participate in activities to increase knowledge	3.278	0.526
12 Engage in artistic activities	3.222	0.671
13 Manage emotions well	3.191	0.825
14 Engage in sports	3.268	0.799
15 Engage in recreational activities	3.165	0.819
16 Build up from frustration	3.042	0.701
17 Keep myself busy	3.153	0.734
18 I will seek the cooperation of parents	2.996	0.743
19 Seek help from friends	2.938	0.762
20 Seek help from experienced people or groups	2.783	0.775
21 Seek help from superiors	2.774	0.764
23 Seek assistance and advice from other teachers	2.864	0.788
24 Seek assistance from professionals outside the school	2.553	0.868

The results of the descriptive statistics showed that the mean values of the coping strategies of the subjects ranged between 2.553 and 3.278, which was a moderate level.

## 4 Discussion

This paper took the kindergarten teachers in Guangzhou and Zhanjiang City as the research object, and analyzed the reliability and validity of the Stress Coping Scale for Early Childhood Teachers by obtained data through the questionnaire, which was divided into 3 dimensions: rational thinking, emotion regulation and support seeking, the internal consistency coefficient of the overall scale was 0.912, the internal consistency reliability of the 3 subscales ranged from 0.862–0.912, with a fair degree of reliability, and in terms of validity, the standardized loading coefficients for the 24 items were in the range of 0.453–0.838 in absolute value, which were greater than 0.4.

For the analysis of discriminant validity, for rational thinking, its AVE square root value is 0.697, which was slightly higher than the maximum value of the absolute value of the inter-factor correlation coefficient of 0.695, and implied that its discriminant validity was still good but there was room for optimization. For emotion regulation and support seeking, their AVE square root values were 0.725 and 0.737, which were greater than the maximum absolute values of the inter-factor correlation coefficients of 0.425 and 0.695, which meant that the two factors had good discriminant validity. In terms of convergent validity, the corresponding CR values of the three factors were greater than 0.7, and the AVE values of the average variance extraction of the three factors were slightly higher than 0.5, implying that their convergent validity was fair.

Combined with the factor loading coefficients in Table 3, the factor loading coefficients of the three items “Thinking about problems from different perspectives,” “Referring to previous experiences,” and “Seeking advice from education experts and scholars” were below 0.5, and the MI index of the item “Keeping myself busy” was greater than 20 for both rational thinking and support seeking dimensions.

According to the results of Wen’s study, the minimum requirement for GFI, NFI, CFI, TLI and IFI is greater than 0.85, the best was greater than 0.9, and the RMSEA was less than 0.10 (Wen and Ye, 2014). The model fitting indices of this scale showed that GFI = 0.866 > 0.85, CFI = 0.914 > 0.9, NFI = 0.862 > 0.85, TLI = 0.905 > 0.9, IFI = 0.915 > 0.9, and RMSEA = 0.066 < 0.10, and the model basically meets the requirements of the measurement. Correlation analysis between the coping strategy scale and the COPE questionnaire, which was the standardized scale, showed that rational thinking, emotion regulation, and support-seeking were significantly and positively correlated with the two dimensions of help-seeking and cognitive coping of the COPE questionnaire, and the correlation coefficients were all greater than 0.4, which indicated that the correlation between this scale and the standardized scale was high.

Low factor loading coefficients indicate a weak relationship between the observed and potential variables, and because of cross loading this usually indicates that the meaning of the measurement item is unclear or not applicable to the current factor analysis model. Removal of these measurement terms can improve the interpretability and validity of the model. Therefore, consider removing measures with low factor loading coefficients and cross loadings. For this reason, we can consider delete the three items “Thinking from different perspectives,” “Referring to previous experiences” and “Seeking advice from education experts and scholars,” which had factor loadings below 0.5, and retaining the remaining 21 items “Keeping yourself busy,” which had cross loadings and keep the remaining 21 items.

## 5 Conclusion

In this research, the descriptive statistics scored higher at participate in activities to increase knowledge, engage in sports, engage in recreational activities and keep theirselves busy, which belong to the dimensions of rational thinking and emotion regulation. The researcher concluded that the Chinese cultural background is more implicit, and Chinese kindergarten teachers who need to ask for help from the organization when encountering difficulties and troubles will be hesitant to ask for help from their colleagues and superiors, worrying that other people will doubt their ability to do their job, and afraid of asking for help from others because of the emotional embarrassment, so they are more inclined think independently to solve their problems and regulate their emotions alone. Based on this, the researcher suggests that the director should try to keep good communication with teachers to understand what they really need. The director should send emails or make phone calls to communicate with the teachers from time to time, and for the difficulties encountered by the teachers, the director and administrators should make reasonable adjustments to the resources and try to help the teachers to solve the problems, in addition, the director and administrators should reasonably distribute the work tasks, and try to be as fair and just as possible to treat each teacher without prejudice against the employees. Secondly, directors and administrators should trust teachers and believe in their competence in the classroom, and when they encounter complaints from parents, they should first fully understand the situation instead of blaming the teachers. Increasing mutual trust between leaders and staff is an effective way to improve teachers’ stress coping level. Kindergartens can also arrange some sports and recreational activities for teachers. Appropriate physical activities can reduce stress levels, increase communication among teachers, and establish a good working environment, all of which are useful for improving teachers’ stress coping levels.

There were still some deficiencies in this study, which could be further improved by the subsequent researchers. In terms of the research object, only early childhood teachers in Guangzhou City and Zhanjiang City were selected as the research object in this study, and there was lack of data on the stress coping strategies of early childhood teachers in other regions; Guangzhou City, as a first-tier city, the education department pays more attention to the mental health and occupational stress of the early childhood teachers perhaps slightly more than that in other cities, which may affect the result of the coping strategies. Secondly, the scale still has the problem of cross loading in some items, which is recommended to be deleted in this study, and the researcher may consider retesting the reliability and validity of the revised scale in the subsequent study, which may be due to the fact that the amount of data is still not large enough, and needs to be further increased, or it may be due to the fact that the design of the items is still insufficient and needs to be further adjusted. In subsequent studies, researchers can collect data on the occupational stress coping strategies of early childhood teachers in different regions for repeated measurements to further improve the scale and enrich the empirical research data in the field of preschool education.

## Data Availability

The raw data supporting the conclusions of this article will be made available by the authors, without undue reservation.

## References

[ref1] Bian J. (2018). Han research on the relationship between occupational stress sources, attribution styles and their coping strategies of early childhood teachers. Lanzhou: Northwest Normal University.

[ref2] Cai Y. (2021). Causes of burnout and coping strategies of early childhood teachers. Enlight. Wisdom 7:11.

[ref3] Cao Q. Hao H. Sabitha R. Vadivel T. (2021). Occupational stress management of college English teachers under flipped classroom teaching model. Aggress. Violent Behav. 11, –101712. doi: 10.1016/j.avb.2021.101712

[ref4] Cao K. Yang N. (2022). The relationship between occupational stress and burnout among early childhood teachers: the moderating role of psychological resilience. Basic Educ. Ref. 11, 34–39.

[ref5] Carver-Thomas D. Darling-Hammond L. (2017). Teacher turnover: why it matters and what we can do about it. Learn. Policy Inst. 8, 1–60.

[ref6] Darius S. Hohmann C. B. Siegel L. Böckelmann I. (2021). Relationship between burnout risk and individual stress processing strategies in early childhood teachers. Psychother. Psychosom. Med. Psychol. 71, 230–236. doi: 10.1055/a-1376-6962, PMID: 33682917

[ref7] Friedman-Krauss A. H. Raver C. C. Morris P. A. Jones S. M. (2014). The role of classroom-level child behavior problems in predicting preschool teacher stress and classroom emotional climate. Early Educ. Dev. 25, 530–552. doi: 10.1080/10409289.2013.817030

[ref8] Lazarus R. S. Folkman S. (1984). Stress, appraisal, and coping. New York: Springer Publishing Company.

[ref9] Lee M.-T. (2018). Analysis of work stress and coping strategies of early childhood teachers--an example of kindergarten in Anning, Yunnan. J Northwest Coll. Adult Educ. 3, 106–113.

[ref10] Li Y. (2022). A study on the prediction of kindergarten parent-teacher relationship on teacher-child relationship: The mediating role of parental involvement. Changchun: Northeast Normal University.

[ref11] Liu Y. T. (2022). A study on the prediction of kindergarten parent-teacher relationship on teacher-child relationship: The mediating role of parental involvement: Northeast Normal University.

[ref12] Sheng M. Q. (2020). Survey and research on the current situation of occupational stress of elementary school beginning teachers. Shanghai: Shanghai Normal University.

[ref13] Song Z. J. (2019). An analysis of the current situation of emotional labor and countermeasures of early childhood teachers. Road Success 1:36.

[ref14] Tseng Y. (2018). A study on the work stress and coping strategies of teachers in integrated elementary school classrooms. Unpublished master’s thesis,. Taitung: Department of Special Education, National Taitung University.

[ref15] Wang F. (2022). A study on occupational stress, social support, subjective well-being and correlation among secondary school physical education teachers in Chengdu. Chengdu: Sichuan Normal University.

[ref16] Wang J. (2023). A study of the relationship between occupational stress, supervisor support and emotional labor among early childhood teachers. J. Northwest Coll. Adult Educ. 3, 28–33.

[ref17] Wang J. L. Shen P. F. Deng J. J. (2022). Occupational stress and burnout among early childhood teachers in Inner Mongolia Autonomous Region: the mediating effect of occupational identity. Campus Psychol. 20, 270–275.

[ref18] Wen C. L. Ye B. J. (2014). Mediation effects analysis: methodology and model development. Adv. Psychol. Sci. 22:731.

[ref19] Wong S. R. (2004). A study of kindergarten teachers’ work stress and coping strategies. Tainan: National Tainan University.

[ref20] Wu Y. R. (2023). An empirical study on the relationship between career stress and career commitment of novice kindergarten teachers. Jincheng: Shaanxi University of Technology.

[ref21] Xiao J. H. Xu X. F. (1996). A study of the validity and reliability of the coping styles questionnaire. Chin. J. Ment. Health 4, 164–168.

[ref22] Xiong Q. (2008). A study on the prediction of self-regulation on the relationship between daily stress and coping strategies. Beijing: Capital Normal University.

[ref23] Yang X. C. (2021). Kindergarten teachers’ measures to cope with work stress. New Curric. Res. 35, 119–120.

[ref24] Zeng W. L. (2022). The effects of occupational stress on psychological resilience in early childhood teachers: A mediated moderation model. Nanchang: Nanchang University.

[ref25] Zhang Y. N. (2024). A study of the effect of stress perception on kindergarten teachers’ job satisfaction. Baoding: Hebei University.

